# Supplementary material for: Evidence of Coulomb liquid phase in few-electron droplets
Source: Nature. 2025 Jun 25;642(8069):928–33. doi: 10.1038/s41586-025-09139-z (PMC12221981; doi:10.1038/s41586-025-09139-z)
Supplement: Supplementary file 1 — This file contains Supplementary Notes, Supplementary Figs. 1–8, Supplementary Tables 1 and 2 and Supplementary References [file 41586_2025_9139_MOESM1_ESM.pdf]

nature portfolio

https://doi.org/10.1038/s41586-025-09139-z

Supplementary information

Evidence of Coulomb liquid phase in few-electron droplets

In the format provided by the authors and unedited

SUPPLEMENTARY INFORMATION

for

Evidence of Coulomb liquid phase in few-electron droplets

Jashwanth Shaju, Elina Pavlovska, Ralfs Suba, Junliang Wang,
Seddik Ouacel, Thomas Vasselon, Matteo Aluffi, Lucas Mazzella,
Clément Geffroy, Arne Ludwig, Andreas D. Wieck, Matias Urdampilleta,
Christopher Bäuerle, Vyacheslavs Kashcheyevs, and Hermann Sellier

Contents

1. CONTROL OF MULTI-ELECTRON TRANSFER 2
   1.1. Preparation and sending procedure 2
   1.2. Placing all electrons in the same SAW minimum 2
   1.3. Placing all electrons in different SAW minima 3
   1.4. Detection of multiple electrons 6

2. EFFECTIVE TEMPERATURE IN THE CENTRAL CHANNEL 7
   2.1. Partitioning versus barrier-gate voltage for one electron 7
   2.2. Extraction of transition rates with two-site master equations 7
   2.3. Thermally-activated hopping in a quartic double-well potential 9
   2.4. Discussion of the effective temperature 10

3. STATISTICAL INDISTINGUISHABILITY OF S1 AND S2 ELECTRONS 12
   3.1. Selection of a suitable barrier-gate voltage 12
   3.2. Comparison of different loading configurations 13

4. RECONSTRUCTION FORMULA FOR INDEPENDENT ELECTRONS 14

5. COUNTING STATISTICS AND CUMULANTS 15
   5.1. Multivariate and univariate cumulants 15
   5.2. Relation between multivariate cumulants and full counting statistics 16
   5.3. Comparison of different types of cumulants 17

6. ANALYSIS OF 2e/2e PARTITIONING DATA 19

1. CONTROL OF MULTI-ELECTRON TRANSFER

To prepare a droplet of several electrons, precise control over electron placement in a selected potential minimum of the surface acoustic wave (SAW) is essential. This task is not straightforward and several parameters must be meticulously tuned and optimised. The procedure is described here.

1.1. Preparation and sending procedure

First, the two source quantum dots (QD) are initialised with the desired number of electrons, while the two detector QDs are left empty. The number of electrons in each QD is identified with the help of their respective loading map as shown in Fig. S1a. This map is obtained by measuring the current change through the quantum point contact (QPC) coupled to the QD, in a reference configuration of the gate voltages. This charge sensing method provides a very accurate count of the QD electrons, as illustrated in Fig. S1b. Each pixel in the loading map represents the change in QPC current after a specific loading process controlled by the voltages V_{R} and V_{C} applied to the reservoir gate (R) and channel gate (C) of the source QD [1].

Then, the system is configured to a holding state, in which the loaded electrons remain trapped in the QDs until the SAW arrives. The yellow regions of the holding maps in Fig. S1c,d represent the various sets of gate voltages for which the three electrons initially loaded in the source QD remain trapped. The holding configuration for SAW transport is when V_{R} is around $-1.2\,\mathrm{V}$.

About one microsecond after the SAW is launched at the IDT, the SAW train reaches the source QDs and the prepared electrons are transported across the device by the piezo-electric potential of the wave. Using ultra-short RF pulses, we can precisely control the electron distribution within the SAW train, with either all electrons in the same potential minimum, or just a subset in the same minimum, or all in different minima. The two main configurations are detailed below.

1.2. Placing all electrons in the same SAW minimum

We discuss here the procedure for transporting all the electrons together in the same SAW minimum, thereby forming a single electron droplet. The sending process is configured independently for each source using a single detector (D1 for S1 and D2 for S2). For this purpose, a large detuning voltage Δ is applied to the side gates to tilt the confinement potential of the central channel and direct all the electrons towards the selected detector.

Figure S2a and S2b show the sending and catching maps of the source and detector QDs, when the SAW is not launched. Note that the graph axis correspond to the gate voltages of the *source* QD in both maps. These maps show that no electron is transferred, as expected in absence of SAW.

We then launch a 180 µm-long SAW train (60 ns of RF signal at 3 GHz) and count the final number of electrons in the source and detector QDs to construct the sending and catching maps shown in Fig. S2c and S2d. For gate voltages in the bottom-right sector of the maps, 1, 2, or 3 electrons have been transferred as indicated by the specific amplitude of the QPC

current change. However, the electrons transported in this way are not transferred in the same potential minimum of the SAW train, but in random minima.

To place all the electrons into the same SAW minimum, the gate voltages V_C^{send} and V_R^{send} should be chosen in the top-right sector of the maps, such that the electrons remain trapped in the source QD even when the SAW train passes by. The controlled sending is then achieved by applying an ultra-short negative voltage pulse to the plunger gate (P) of the source QD to rise the energy of the electrons and enable their catching and transport by the SAW, as shown in Fig. S2g and S2h. Applying an RF pulse with a time duration (δt_{trig}) shorter than the SAW period ensures that the electrons are placed in only one SAW minimum. Both the amplitude (V_P) and the time delay (τ_P) of the trigger pulse are carefully adjusted to ensure that the three electrons are injected into the designated SAW minimum [1, 2]. The values V_C^{send} and V_R^{send} are also optimized to get the highest transfer efficiency.

Since sending more than 3 electrons from a single source poses considerable technical challenges, sending 4 or 5 electrons is achieved by using the two sources S1 and S2 which are synchronised via their trigger pulse. For example, a droplet of 5 electrons is obtained by sending 3 electrons from S1 and 2 electrons from S2, all in the same SAW minimum. They are initially in different rails, but later join in the central channel.

1.3. Placing all electrons in different SAW minima

To investigate the partitioning of uncorrelated electrons, we have to place the electrons in different SAW minima. This configuration can be achieved by a combination of random and triggered electron sending, as well as a combination of the two electron sources S1 and S2. For the case of three electrons, we can for example proceed in the following ways.

A first possibility is to load three electrons in a single source and simply apply a SAW train without triggering the plunger gate. In this case, V_R^{send} is adjusted to allow the direct sending of the three electrons. When the sending takes place without trigger pulse, the electrons are automatically distributed over different and random SAW minima. This property is evidenced by the absence of correlation in the partitioning experiments performed in this way.

A second possibility is to load three electrons in a single source, as above, but then send two electrons randomly and the third one with a trigger pulse. In this case, V_R^{send} is adjusted to allow the direct sending of only two electrons.

A third possibility is to load two electrons in the source S1 and one electron in the source S2. We can either use the random sending procedure of the two electrons of S1, while the electron in S2 is sent using a trigger pulse at a delayed time near the end of the SAW train, or send the first electron of S1 randomly and the second electron of S1 with a trigger pulse, while the electron of S2 is sent with a trigger pulse at a later time.

All these procedures ensure that the three electrons are in three different SAW minima.

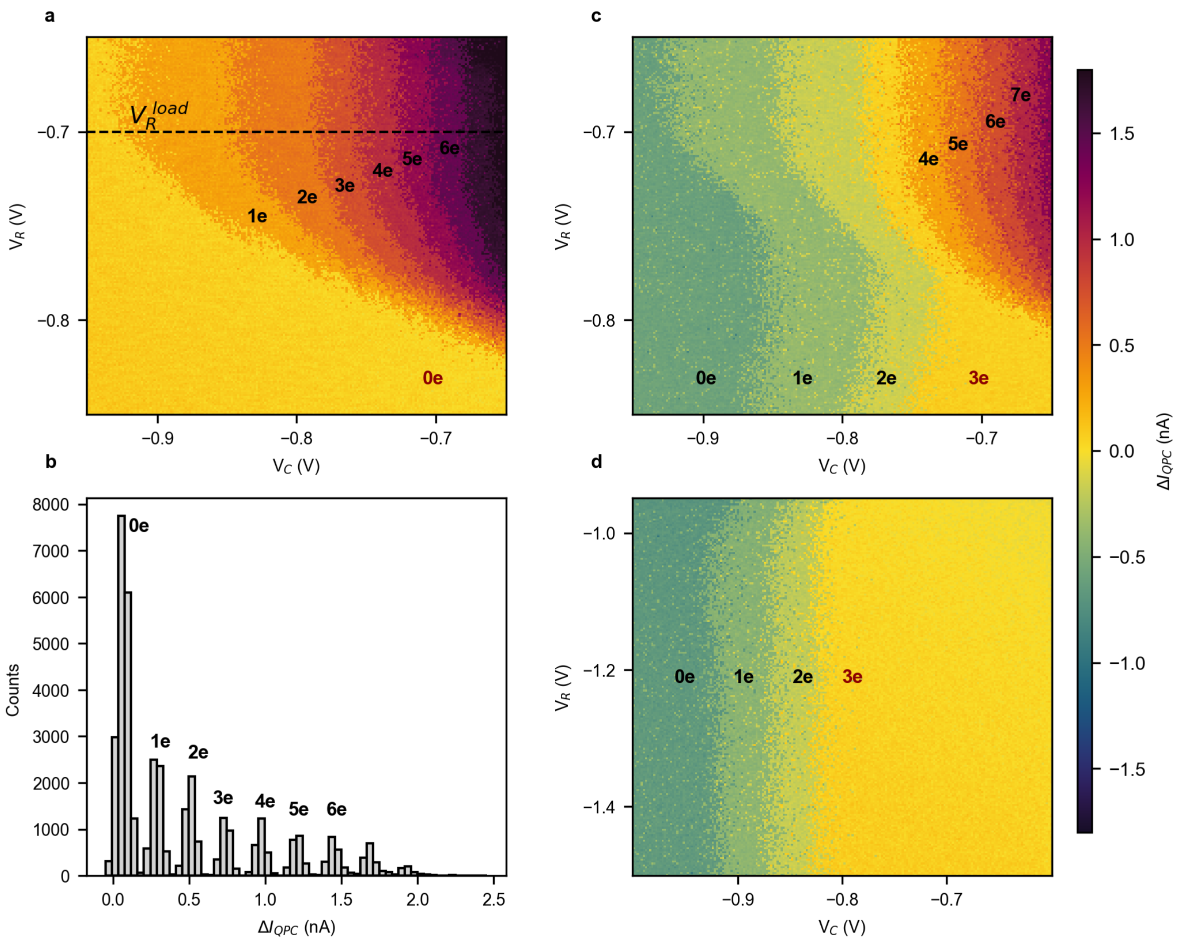

Fig. S1. **Loading and holding maps of a source quantum dot.** **a**, Loading map of the electron source S1. The axis V_{R} and V_{C} denote the reservoir (R) and channel (C) gate voltages of the source QD. Each point on the map shows the change in the current through the charge sensor (QPC) after sweeping the gates from a reference position to the gate values of that point and returning to the reference position. Each colour corresponds to a different number of electrons in the QD. The initial electron number before the sweep is zero ($N^{\mathrm{init}} = 0$). The dotted line at $V_{\mathrm{R}}^{\mathrm{load}} = -0.70\,\mathrm{V}$ marks the set-point value for electron loading. **b**, Histogram of QPC currents from the map in **a**, showing well-separated peaks. **c**, Same map as in **a** but for an initial electron number $N^{\mathrm{init}} = 3$. Dotted lines delimit the region (yellow) where the 3 electrons remain trapped in the dot. This map can be viewed as a holding map. **d**, Holding map for $N^{\mathrm{init}} = 3$ in the gate range of the sending configuration.

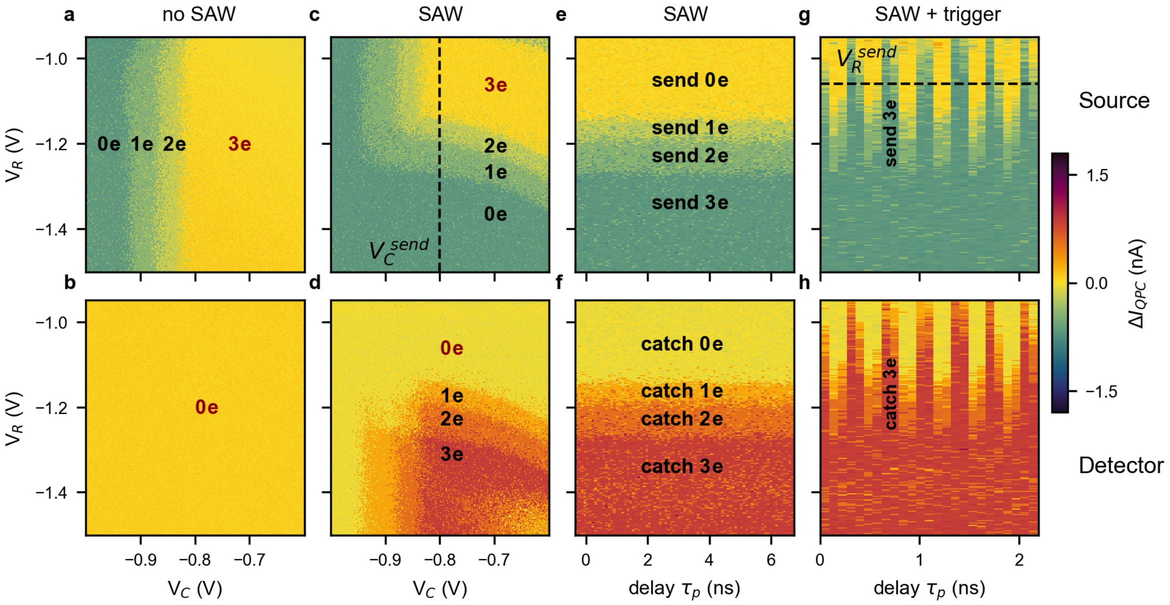

Fig. S2. **Controlled sending of $N = 3$ electrons.** Top panels: Sending maps of the source S1. Bottom panels: Catching maps of the detector D2. The side gates of the central channel are adjusted to tilt the transverse potential well and direct all the electrons toward D2. Three electrons have been initially loaded into the source QD. The voltage V_{R} is then set to a more negative value, to prevent electrons from escaping the dot towards the reservoir when the SAW arrives. **a,b**, Maps before applying the SAW, with all 3 electrons remaining in the source (yellow) and no electron in the detector (yellow). **c,d**, Maps after applying the SAW, showing that 1, 2, or 3 electrons are transferred from the source (green) to the detector (orange). **e,f**, Maps with the SAW applied, at a fixed voltage $V_{\mathrm{C}}^{\mathrm{send}} = -0.80\,\mathrm{V}$ (working point for sending), recorded without trigger pulse in a control experiment to check the electron sending as a function of V_{R}. **g,h**, Maps with the SAW applied, and with a trigger pulse applied on the plunger gate (P) at different time delays τ_{P}. Simultaneous sending of 3 electrons is achieved with a short negative pulse having $V_{\mathrm{P}} = -0.50\,\mathrm{V}$ and $\delta t_{\mathrm{trig}} = T_{\mathrm{SAW}}/4 \approx 90\,\mathrm{ps}$. The dotted line indicates the working point $V_{\mathrm{R}}^{\mathrm{send}} = -1.06\,\mathrm{V}$ used to trigger the sending of 3 electrons.

1.4. Detection of multiple electrons

For partitioning experiments with N electrons, the gate voltages of the detector QDs are carefully tuned to enable the catching of up to N electrons. Figure S3 shows the histograms of current changes in the QPC sensors attached to the two detectors (D1 and D2) for the partitioning data of $N = 5$ electrons in the same SAW minimum. The presence of well-separated peaks demonstrate the detector capability of catching up to 5 electrons.

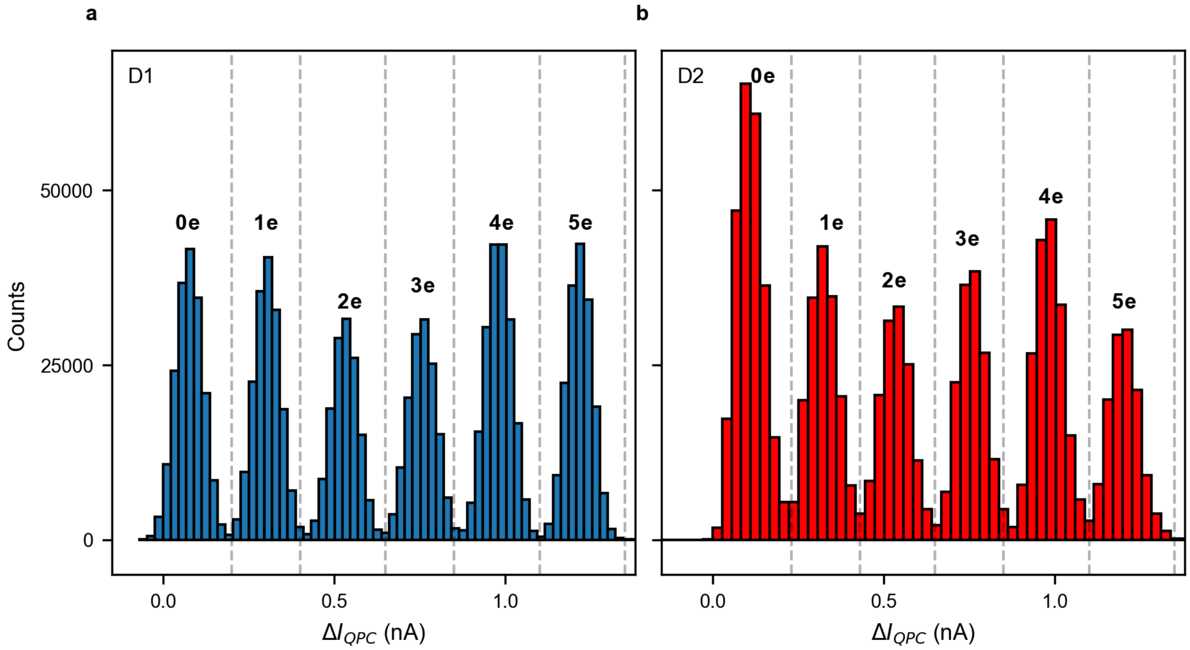

Fig. S3. **Multi-electron detection.** Histograms of the QPC current changes for (**a**) detector D1 and (**b**) detector D2, during the partitioning of $N = 5$ electrons within the same SAW minimum (data collected for a full sweep of side-gate detuning before post-selection of residual transfer errors). This illustrates the capability to detect up to 5 interacting electrons.

2. EFFECTIVE TEMPERATURE IN THE CENTRAL CHANNEL

In our experiment, the electron droplet is confined in three dimensions by the combined influence of the heterostructure band diagram (growth direction), the moving piezoelectric potential induced by the SAW (longitudinal direction) and the electrostatic potential induced by the surface gates (transverse direction). In the central channel, the presence of a narrow barrier gate in-between the two side gates introduces a small barrier in the middle of the transverse confinement potential. In this section, we discuss the impact of the barrier-gate voltage V_{B} on electron partitioning at the Y-junction, and use this adjustable parameter to estimate the effective electron temperature with a model of thermally activated hopping in a double-well potential.

2.1. Partitioning versus barrier-gate voltage for one electron

With side-gate voltages held constant around zero detuning to have symmetric partitioning, we measured the partitioning probability as function of the barrier-gate voltage V_{B} for a single electron sent from source S1 or from source S2 (Fig. S4a). At $V_{\mathrm{B}} = -1.5\,\mathrm{V}$, the upper and lower rails are fully isolated, preventing electron from transitioning from one side to the other. For $V_{\mathrm{B}} \geq -1.25\,\mathrm{V}$, electrons sent from sources S1 and S2 show the same partitioning result $P^{\mathrm{S1}}_{(0,1)} = P^{\mathrm{S2}}_{(0,1)}$ and hence are statistically indistinguishable. This property is the consequence of the very long (40 µm) central channel which gives the electron enough time (14 ns) to equilibrate between the two rails by tunneling or hopping through the barrier.

For all partitioning experiments reported in this work, the barrier-gate voltage has been fixed at $V^0_{\mathrm{B}} = -1.25\,\mathrm{V}$, such that both sources S1 and S2 can be used indifferently, with $P^{\mathrm{S1}}_{(N-n,n)} = P^{\mathrm{S2}}_{(N-n,n)}$, simply noted $P_{(N-n,n)}$ (for supporting data, see Supplementary Note 3).

2.2. Extraction of transition rates with two-site master equations

To analyse the single-electron partitioning data of Fig. S4a, the double-well potential of the central channel is first modelled by a simplified two-site system, which will be refined later with a 1D quartic potential.

We model the single-electron partitioning data with the two-site master equations

$$\partial_t \begin{pmatrix} P_{\mathrm{U}} \\ P_{\mathrm{L}} \end{pmatrix} = \begin{pmatrix} -\Gamma_{\mathrm{U}\to\mathrm{L}} & \Gamma_{\mathrm{L}\to\mathrm{U}} \\ \Gamma_{\mathrm{U}\to\mathrm{L}} & -\Gamma_{\mathrm{L}\to\mathrm{U}} \end{pmatrix} \begin{pmatrix} P_{\mathrm{U}} \\ P_{\mathrm{L}} \end{pmatrix}, \tag{S.1}$$

where the time-independent transition rates $\Gamma_{\mathrm{U}\to\mathrm{L}}$ and $\Gamma_{\mathrm{L}\to\mathrm{U}}$ correspond to stochastic single-electron transitions from the upper to the lower rail (and vice-versa). Solving this system of coupled linear equations, two solutions for $P_{\mathrm{U}}(t)$ are obtained corresponding to two different initial conditions: electron sent from S1 ($P^{\mathrm{S1}}_{\mathrm{U}}(0) = 1$) and electron sent from S2 ($P^{\mathrm{S2}}_{\mathrm{U}}(0) = 0$). The experimentally measured probabilities $P_{(0,1)}$ correspond to these solutions at $t = \tau$, where τ is the duration of the electron flight along the central channel,

$$\begin{aligned} P^{\mathrm{S1}}_{\mathrm{U}}(\tau) &= \frac{\Gamma_{\mathrm{L}\to\mathrm{U}} + \Gamma_{\mathrm{U}\to\mathrm{L}}\, e^{-(\Gamma_{\mathrm{U}\to\mathrm{L}}+\Gamma_{\mathrm{L}\to\mathrm{U}})\tau}}{\Gamma_{\mathrm{U}\to\mathrm{L}} + \Gamma_{\mathrm{L}\to\mathrm{U}}}, \\ P^{\mathrm{S2}}_{\mathrm{U}}(\tau) &= \frac{\Gamma_{\mathrm{L}\to\mathrm{U}} - \Gamma_{\mathrm{L}\to\mathrm{U}}\, e^{-(\Gamma_{\mathrm{U}\to\mathrm{L}}+\Gamma_{\mathrm{L}\to\mathrm{U}})\tau}}{\Gamma_{\mathrm{U}\to\mathrm{L}} + \Gamma_{\mathrm{L}\to\mathrm{U}}}. \end{aligned} \tag{S.2}$$

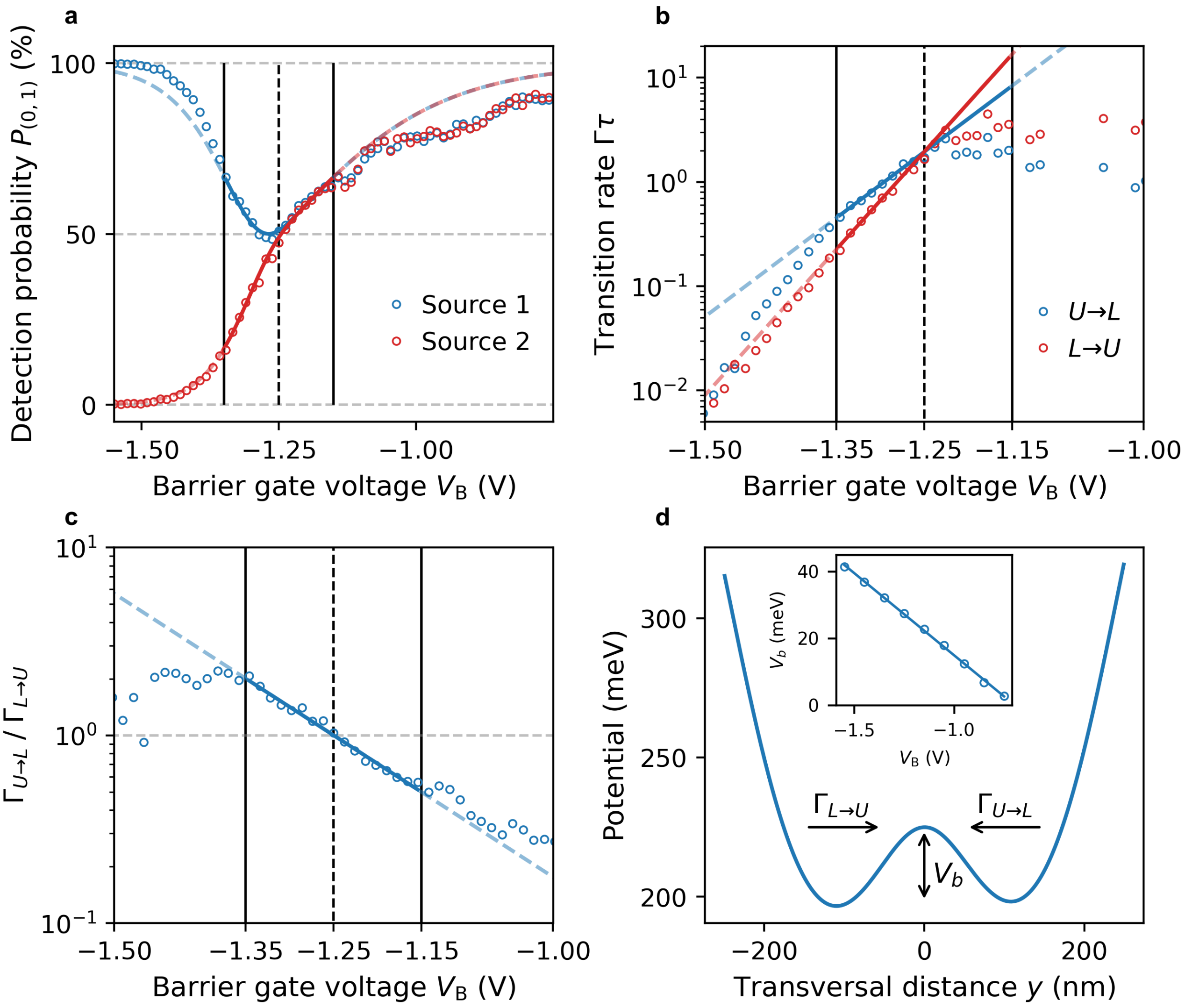

Fig. S4. **Transition rates across the central-channel barrier.** **a**, Detection probabilities P_U^{S1} and P_U^{S2} in the upper detector D1 for single-electron partitioning as function of the barrier-gate voltage V_B and for fixed side-gate voltages $V_U = -1.108\,V$ and $V_L = -1.098\,V$. The electron is sent either from the source S1 or from the source S2. Blue and red solid lines correspond to the two-site model fitted to the experimental data within the fit window around the working point at $V_B^0 = -1.25\,V$, while dashed lines show the model outside the fit window (indicated by the vertical lines). **b**, Transition rates calculated from the experimentally measured detection probabilities after fitting with the two-site model. **c**, Ratio of transition rates for the two directions. **d**, Electrostatic simulation of the potential energy along the transverse direction at V_B^0. The inset shows the barrier-height dependence on barrier-gate voltage.

By inverting these relations, the transition rates $\Gamma_{\mathrm{L(U)\to U(L)}}$ can be expressed in terms of the experimentally measured probabilities, as shown in Fig. S4b. In the vicinity of the working point V_B^0 selected for the partitioning experiments, the transition rates can be approximated as exponentially dependent on the barrier-gate voltage. For voltages above V_B^0, the characteristic transition times $1/\Gamma_{\mathrm{L(U)\to U(L)}}$ are much shorter than the duration of the flight in the central channel, giving rise to statistical equilibrium between the two rails. The two electron exchange rates are not equal, and their ratio plotted in Fig. S4c is exponentially dependent on V_B.

Based on these observations, we express the rates as

$$\begin{aligned}\Gamma_{\mathrm{U\to L}} &= \Gamma_0\, e^{c_\mathrm{B}^{\mathrm{U\to L}}(V_\mathrm{B}-V_\mathrm{B}^0)}\\ \Gamma_{\mathrm{L\to U}} &= \Gamma_0\, e^{c_\mathrm{B}^{\mathrm{L\to U}}(V_\mathrm{B}-V_\mathrm{B}^0)}\end{aligned} \tag{S.3}$$

and fit the measured probabilities with the solutions (S.2) together with the expressions (S.3) as shown by solid lines in Fig. S4a. The model agrees well with experimental data within the fitting window and predicts qualitatively the measured probabilities outside the window. The best fit parameters are $c_\mathrm{B}^{\mathrm{U\to L}} = 14.6\,\mathrm{V}^{-1}$, $c_\mathrm{B}^{\mathrm{L\to U}} = 21.5\,\mathrm{V}^{-1}$ and $\Gamma_0\tau = 1.94$. The difference between the two slope coefficients c_B corresponds to an asymmetric influence of the barrier gate on the electrostatic potential of the two sites. The inferred value of $\Gamma_0\tau$ implies that at the working point V_B^0 the single-electron probabilities have equilibrated to within 2%.

2.3. Thermally-activated hopping in a quartic double-well potential

The above barrier-gate dependence of the transition rates can be used to extract quantitative information about the energy properties of the electron in the central channel. For this purpose, we consider a simple 1D double-well potential in form of a quartic polynomial, keeping the conventional notation [3],

$$V(y) = V_b + \mu_q\frac{y}{y_0} - 8V_b\frac{y^2}{y_0^2} + 16V_b\frac{y^4}{y_0^4}, \tag{S.4}$$

where V_b is the energy barrier (controlled by the barrier-gate voltage V_B) and μ_q is the energy detuning (controlled by the side-gates voltage difference $\Delta = V_\mathrm{U} - V_\mathrm{L}$). Tuned to symmetry, the potential has two minima at $y = \pm y_0/2$ which we identify with the two sites of the kinetic model considered in previous section. Note that the same potential is used also for multi-electron simulations described in the Methods.

The theory of transition rates [4, 5] generically predicts two regimes that depend on the temperature T: tunneling for $T < T_0$ and hopping for $T > T_0$, where T_0 is the tunneling-to-hopping crossover temperature determined by the curvature of $V(y)$ at the top of the barrier. For the quartic potential, it is given by $k_BT_0 = 3\hbar\omega_y/16$ in terms of the curvature $\omega_y = \sqrt{32V_b/(my_0^2)}$ at the minima of the double-well potential ($m = 0.067m_e$ for electrons in GaAs).

In the regime of thermally-activated hopping, but still in the high barrier limit $\beta V_b \gg 1$ where $\beta = 1/k_\mathrm{B}T$, the temperature-dependent hopping rates (for weak detuning μ_q) can be

approximated as [6]

$$\Gamma_{\mathrm{U}\to\mathrm{L}} = \Gamma_0^{(\mathrm{cl})}\, e^{-\beta(V_b-\mu_q/2)},$$
$$\Gamma_{\mathrm{L}\to\mathrm{U}} = \Gamma_0^{(\mathrm{cl})}\, e^{-\beta(V_b+\mu_q/2)}. \tag{S.5}$$

where the prefactor $\Gamma_0^{(\mathrm{cl})}$ is the classical attempt rate for hopping. These relations can be used to extract the effective electron temperature T in the central channel from the barrier-gate dependence of the experimental transition rates obtained in the previous section. However, to determine the effective temperature in Kelvin, we need the conversion factor between the barrier-gate voltage V_{B} and the barrier energy V_b.

We estimate the numerical values of the parameters of the quartic potential Eq. (S.4) using NextNano [7] self-consistent 3D simulations of the electrostatic potential induced by the surface gates in the modulation-doped GaAs/AlGaAs heterostructure, including non-linear screening by the 2DEG in non-depleted regions. For the actual values of voltages applied to the side gates and barrier gate, the central channel is depleted and forms a double-well potential (Fig. S4d). The potential profile has been simulated for a range of experimental voltages V_{B} in the symmetric configuration $V_{\mathrm{U}} = V_{\mathrm{L}}$ corresponding to $\mu_q = 0$. The energy barrier height V_b (energy difference between the central maximum and the lateral minima) is shown in the inset as function of V_{B}. At the working point $V_{\mathrm{B}}^0 = -1.25\,\mathrm{V}$, the barrier height is $V_b = 27.5\,\mathrm{meV}$ and the distance between the two minima is $y_0 = 220\,\mathrm{nm}$, which gives a tunneling-to-hopping crossover temperature $k_{\mathrm{B}}T_0 = 0.85\,\mathrm{meV}$ (about $10\,\mathrm{K}$). The inset also indicates a linear relationship between the experimental voltage V_{B} and the energy barrier height V_b expressed as

$$V_b = \alpha_{\mathrm{B}}(V_{\mathrm{B}} - V_{\mathrm{B,flat}}) \tag{S.6}$$

with a lever-arm factor $\alpha_{\mathrm{B}} = -49.2\,\mathrm{meV\,V^{-1}}$ and a flat-well voltage $V_{\mathrm{B,flat}} = -0.70\,\mathrm{V}$. Thanks to this linear dependence, the exponents of the hopping rates in Eq. (S.5) can be compared with those of Eq. (S.3) to get the relation

$$\beta|\alpha_{\mathrm{B}}| = \bar{c}_{\mathrm{B}} \tag{S.7}$$

where the coefficient $\bar{c}_{\mathrm{B}} = (c_{\mathrm{B}}^{\mathrm{U}\to\mathrm{L}} + c_{\mathrm{B}}^{\mathrm{L}\to\mathrm{U}})/2 = 18\,\mathrm{V^{-1}}$ is the average of the two fitted coefficients. The effective electron temperature in the moving confinement potential is then obtained as

$$k_{\mathrm{B}}T \equiv \beta^{-1} = \frac{|\alpha_{\mathrm{B}}|}{\bar{c}_{\mathrm{B}}} = 2.73\,\mathrm{meV}\ (32\,\mathrm{K})\,. \tag{S.8}$$

The value $T > T_0$ confirms the consistency of our assumptions for Eq. (S.5) that electron exchanges between the two wells takes place in the classical hopping regime.

2.4. Discussion of the effective temperature

The electrostatic simulation also provides the detuning lever arm factor $\alpha = 0.16\,\mathrm{meV\,mV^{-1}}$, which is introduced in the main text to relate the energy detuning μ of the Ising model to the side-gates voltage difference by $\mu = -\alpha(\Delta - \Delta_0)$. The global fitting parameter $\alpha/k_{\mathrm{B}}T = 0.064\,\mathrm{mV^{-1}}$ obtained by fitting the multi-electron partitioning data with the Ising model (see Extended Data Table 1) can therefore provide another estimate of the effective

electron temperature, and we get $k_\mathrm{B}T = 2.5\,\mathrm{meV}$ (29 K) in good agreement with the value obtained in the previous section.

The interaction strength U of the Ising model can be then deduced from the second fitting parameter $U/k_\mathrm{B}T$. We find that U decreases from 3.2 to 1.8 meV when N increases from 2 to 5 (screening of Coulomb repulsion in the droplet). At the same time, the Néel temperature T_N increases from 37 to 53 K.

The values of the effective temperature estimated above are in reasonable agreement with $T = 25\,\mathrm{K}$ found from matching the observed multi-electron correlations to Monte-Carlo simulations, as described in the main text. The latter also gives an independent way to estimate the level-arm factor α resulting in values within the range of 0.13 to $0.15\,\mathrm{meV\,mV^{-1}}$, consistent with $0.16\,\mathrm{meV\,mV^{-1}}$ estimated from electrostatic simulations.

Nevertheless, one has to exercise caution in interpreting the hopping-model estimates, since they only include single-electron hopping between two sites with a constant effective rate during the propagation time τ before reaching the Y-junction. The two-site hopping model averages over the disorder in the central channel and does not take into account the excitation of the electrons as they move along the not perfectly uniform potential landscape [1]. We attribute the high effective temperature of the electron droplet to this physical effect. Additionally, non-equilibrium dynamics of electrons on a scale much shorter than τ during partitioning [8] at the exit Y-junction may also contribute to the freeze-out temperature estimated in the sudden quench approximation.

3. STATISTICAL INDISTINGUISHABILITY OF S1 AND S2 ELECTRONS

To prepare an electron droplet with $N > 3$, we utilise both the S1 and S2 sources and synchronise them to place all the electrons into the same SAW minimum. However, we must ensure that the electrons injected from sources S1 and S2 are statistically indistinguishable. This is accomplished by first tuning the barrier height of the central channel to achieve indistinguishability for $N = 1$ electron, and then checking the indistinguishability in the partitioning experiments for $N = 2$ to 4 electrons.

3.1. Selection of a suitable barrier-gate voltage

A single electron is injected from either source S1 or source S2, and the partitioning probability is recorded as a function of the barrier-gate voltage V_{B}, with the side gates kept fixed at three different detuning values, as shown in Fig. S5.

For $V_{\mathrm{B}} < -1.50\,\mathrm{V}$ in panel b (near zero detuning) or $V_{\mathrm{B}} < -1.60\,\mathrm{V}$ in panels a and c (for large detuning), a high barrier prevents the electron from hopping between the two wells of the transverse double-well potential, such that the two wells are fully isolated.

For $V_{\mathrm{B}} \geq -1.25\,\mathrm{V}$, the partitioning probabilities are identical for electron injections from source S1 and from source S2. The operating point $V_{\mathrm{B}}^0 = -1.25\,\mathrm{V}$ was selected to have electron indistinguishability, while maintaining a strong confinement within the central channel. At this barrier-gate voltage, an electron injected from source S1 is statistically equivalent to an electron injected from source S2, for all the detuning voltages used in the partitioning experiments.

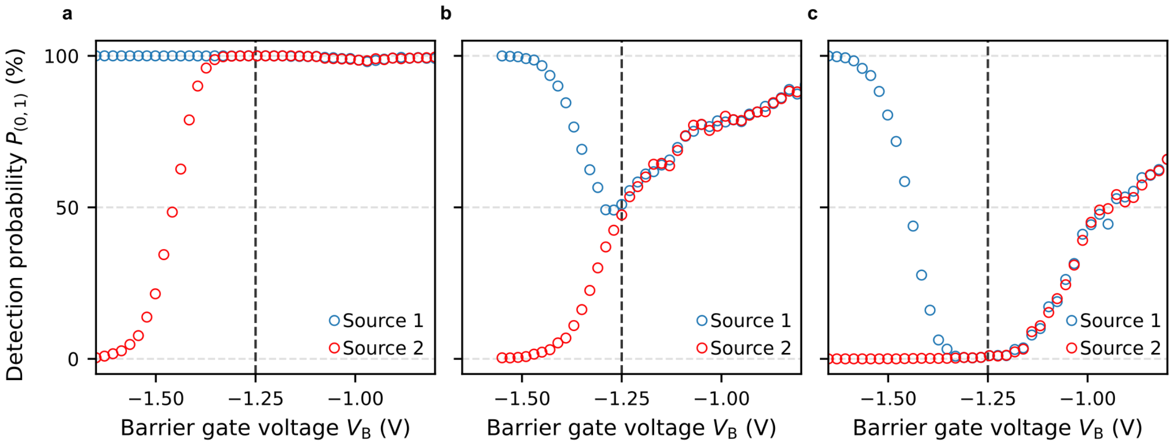

Fig. S5. **Barrier-gate dependence and electron indistinguishability.** Partitioning probability of a single electron as function of the barrier-gate voltage V_{B}. The blue circles represent injection from the upper source S1, while the red circles indicate injection from the lower source S2. Each data point is based on 3,000 single-shot measurements and represents the probability $P_{(0,1)}$ to detect the electron in the upper detector D1. The vertical dashed line indicates the operating point $V_{\mathrm{B}}^0 = -1.25\,\mathrm{V}$. **a**, The side gates are set to the large positive detuning $\Delta = 85\,\mathrm{mV}$. **b**, The side gates are set to the near-zero detuning $\Delta = -10\,\mathrm{mV}$ where a symmetric partitioning is achieved at V_{B}^0. **c**, The side gates are set to the large negative detuning $\Delta = -85\,\mathrm{mV}$.

3.2. Comparison of different loading configurations

Here we compare the partitioning of multi-electron droplets created from different configurations of electron number in sources S1 and S2. Fixing the barrier-gate voltage at the operating point V_{B}^{0}, we record the partitioning probabilities of droplets with $N = 2$ to 4 electrons, using different distributions of the N electrons between the two sources. Figure S6 shows that the probabilities are identical regardless of the electron loading configuration. This observation experimentally confirms that, during their evolution along the central channel, the electrons of the droplet have lost information about their source, becoming statistically indistinguishable.

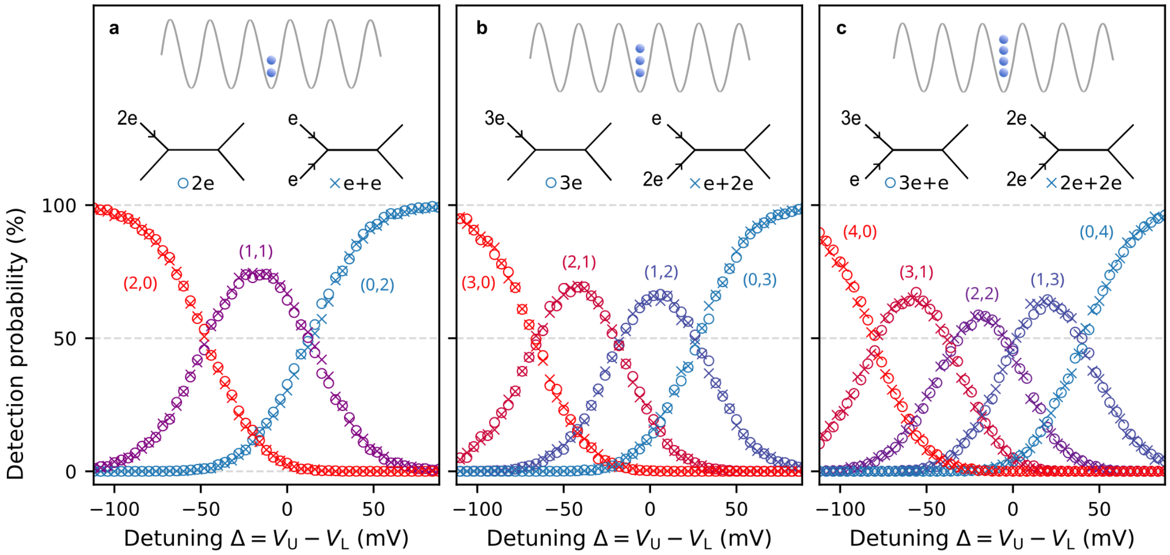

Fig. S6. **Partitioning of electron droplets with different loading configurations.** The barrier-gate voltage is set at the same working point V_{B}^{0} as in the experiments of the main text. The illustration at the top of each panel depicts the loading configurations. The two sources S1 and S2 are synchronised to load all electrons into the same SAW minimum. Each data point is based on 3,000 single-shot measurements. **a**, Partitioning probabilities for an $N = 2$ electron droplet. The configurations are 2e (two electrons from source S1) and e+e (one electron from source S1 and one from source S2). **b**, Partitioning probabilities for an $N = 3$ electron droplet. The configurations are 3e (three electrons from source S1) and e+2e (one electron from source S1 and two from source S2). **c**, Partitioning probabilities for an $N = 4$ electron droplet. The configurations are 3e+e (three electrons from source S1 and one from source S2) and 2e+2e (two electrons from source S1 and two from source S2).

4. RECONSTRUCTION FORMULA FOR INDEPENDENT ELECTRONS

Table S1 explains how to reconstruct the partitioning probabilities of $N > 1$ independent electrons from the knowledge of the $N = 1$ partitioning probabilities. This reconstruction is illustrated in Fig. S7.

e/e	e/e/e	e/e/e/e	e/e/e/e/e
$P_{(2,0)} = P^2_{(1,0)}$	$P_{(3,0)} = P^3_{(1,0)}$	$P_{(4,0)} = P^4_{(1,0)}$	$P_{(5,0)} = P^5_{(1,0)}$
$P_{(1,1)} = 2P_{(1,0)}P_{(0,1)}$	$P_{(2,1)} = 3P^2_{(1,0)}P_{(0,1)}$	$P_{(3,1)} = 4P^3_{(1,0)}P_{(0,1)}$	$P_{(4,1)} = 5P^4_{(1,0)}P_{(0,1)}$
$P_{(0,2)} = P^2_{(0,1)}$	$P_{(1,2)} = 3P_{(1,0)}P^2_{(0,1)}$	$P_{(2,2)} = 6P^2_{(1,0)}P^2_{(0,1)}$	$P_{(3,2)} = 10P^3_{(1,0)}P^2_{(0,1)}$
	$P_{(0,3)} = P^3_{(0,1)}$	$P_{(1,3)} = 4P_{(1,0)}P^3_{(0,1)}$	$P_{(2,3)} = 10P^2_{(1,0)}P^3_{(0,1)}$
		$P_{(0,4)} = P^4_{(0,1)}$	$P_{(1,4)} = 5P_{(1,0)}P^4_{(0,1)}$
			$P_{(0,5)} = P^5_{(0,1)}$

Table S1. Expressions for reconstructing the partitioning probabilities $P_{(N-n,n)}$ of $N = 2$, 3, 4, and 5 non-interacting electrons placed in different SAW minima, using the experimental partitioning probabilities $P_{(1,0)}$ and $P_{(0,1)}$ of a single electron ($N = 1$), as illustrated in Fig. S7.

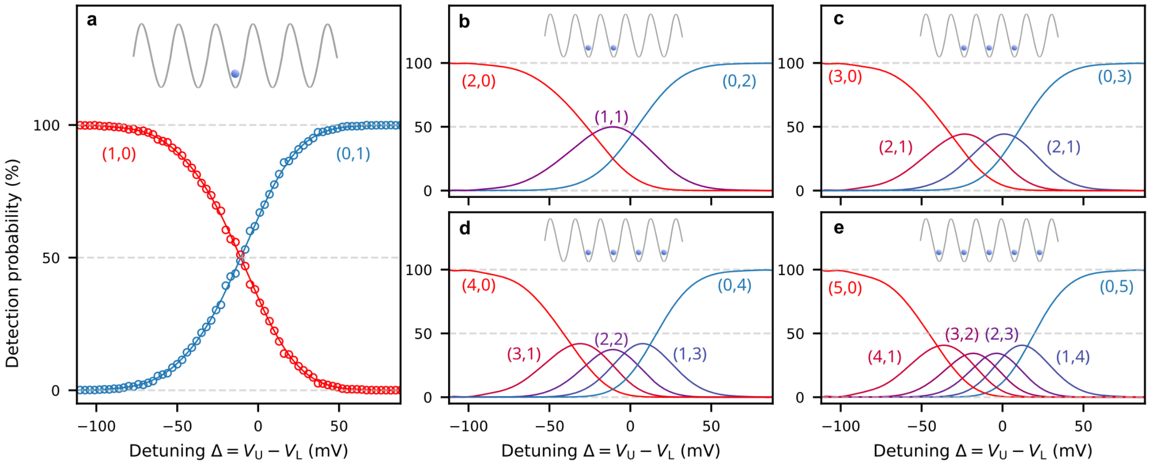

Fig. S7. **Reconstruction of partitioning probabilities of uncorrelated electrons using single-electron data.** **a** Probability distributions for single-electron partitioning as function of side-gates detuning, for $V_B = -1.25$ V. Each data point is based on 3,000 single-shot measurements. Lines are 15th-order polynomial fits, which are used to model multi-electron partitioning probabilities corresponding to uncorrelated electrons distributed among different SAW minima. **b-e**, Reconstruction of the partitioning probabilities for $N = 2$, 3, 4, and 5 uncorrelated electrons placed in different SAW minima, using the expressions given in Table S1.

5. COUNTING STATISTICS AND CUMULANTS

5.1. Multivariate and univariate cumulants

The outcome of the partitioning experiment can be represented by the probabilities p_n to detect n electrons at a chosen detector (here D1), as done in full counting statistics (FCS) [9]. This probability distribution can be characterised in terms of either moments $\langle n^k \rangle$ or cumulants $\langle\langle n^k \rangle\rangle$, which are related to each other by the formal expansion of the appropriate generating function

$$\ln\langle e^{zn}\rangle = \ln\left(\sum_{k=0}^{\infty}\langle n^k\rangle\frac{z^k}{k!}\right) = \sum_{k=1}^{\infty}\langle\langle n^k\rangle\rangle\frac{z^k}{k!} \tag{S.9}$$

where $\langle f(n)\rangle = \sum_n p_n f(n)$ denotes averaging over the probabilities of the FCS. One can express moments through cumulants

$$\langle n^k\rangle = \sum_{j=0}^{k} B_{kj}\left(\langle\langle n\rangle\rangle, \langle\langle n^2\rangle\rangle, \ldots, \langle\langle n^j\rangle\rangle\right) \tag{S.10}$$

and vice versa

$$\langle\langle n^k\rangle\rangle = \sum_{j=1}^{k}(j-1)!(-1)^{j-1}B_{kj}\left(\langle n\rangle, \langle n^2\rangle, \ldots, \langle n^{k-j+1}\rangle\right), \tag{S.11}$$

where B_{kj} are the partial Bell polynomials [10].

Two properties of regular (univariate) cumulants are noteworthy in the context of studying correlations between partitioning events, for an ensemble of N particles. Firstly, that they are additive for independent variables. In our case, the number n of particles detected at D1 can be written as the sum $n = \sum_{j=1}^{N} T_j$ of binary variables $T_j \in \{0, 1\}$ coding the absence (0) or presence (1) of the particle j in the detector D1 for a given realization of the partitioning experiment. If the variables T_j are statistically independent (p_n following a binomial distribution), then $\langle\langle n^k\rangle\rangle_N = N\langle\langle n^k\rangle\rangle_1$. Secondly, if p_n follows Gaussian distribution, $\langle\langle n^k\rangle\rangle = 0$ if $k > 2$. Note that this property is valid only for continuous n, so higher-order ($k > 2$) non-zero cumulants serve as an indication of deviations from Gaussian behaviour but do not distinguish between the effects of discrete n, finite N, or correlations induced by interactions.

The univariate cumulants $\langle\langle n^k\rangle\rangle$ discussed above are therefore not good indicators of inter-particle correlations, as they remain non-zero even when the partitioning is independent. Instead, we consider multivariate cumulants $\langle\langle T_1^{i_1}\ldots T_N^{i_N}\rangle\rangle$ defined with a generating function

$$\ln\langle e^{\mathbf{z}\cdot\mathbf{T}}\rangle = \sum_{i_1,i_2,\ldots,i_N}\langle\langle T_1^{i_1}\ldots T_N^{i_N}\rangle\rangle\frac{z_1^{i_1}\ldots z_N^{i_N}}{i_1!\ldots i_N!} \tag{S.12}$$

where $\mathbf{T} = (T_1, \ldots, T_N)$ and $\mathbf{z} = (z_1, \ldots, z_N)$. The sum $i_1 + i_2 + \ldots + i_N = k$ counts the number of variables T_j (including possible repetitions) involved in the cumulant and represents the order of the cumulant. For a given k, there is one multivariate cumulant for each $\{i_1, ..., i_N\}$ leading to a large number of non-equivalent multivariate cumulants if the variables are not equivalent.

Multivariate cumulants carry more information than univariate cumulants because they capture correlations between multiple variables, allowing for the analysis of many-body systems with correlations between several particles. The key advantage is that a multivariate cumulant between two independent variables T_a and T_b is zero, hence higher-order cumulants reveal the presence of multi-body correlations. In statistics, the multivariate cumulants [11] are also referred to as irreducible correlators, and in field theory, they are known as connected diagrams or connected correlators. Multivariate cumulants and moments are related by general formulas; this relationship is a combinatorial procedure that involves summing over all possible partitions of the indices corresponding to the order k of the cumulant [12]. A more detailed example of applying the general formulas for specific symmetry (determined by electron configuration in SAW minima) is provided in Supplementary Note 6.

5.2. Relation between multivariate cumulants and full counting statistics

FCS measures the cumulative observable $n = \sum_j T_j$ which is symmetric under the permutation of particles. Here we show that knowing the FCS is equivalent to the knowledge of all symmetrised multivariate moments m_k, as expressed by Eq. (2) in Methods. For permutation-symmetric distributions, any multivariate moment or cumulant of k distinct variables depends only on k, hence in the fully symmetric case, the knowledge of m_k implies the knowledge of the corresponding multivariate cumulants κ_k, which can therefore be calculated from the probabilities p_n of the FCS.

We define the fully symmetrised k-th order multivariate moments m_k as an average over all distinct combinations of k variables out of N

$$m_k = \binom{N}{k}^{-1} \langle e_k(T_1, \ldots, T_N) \rangle \tag{S.13}$$

where $\binom{N}{k} = \frac{N!}{k!(N-k)!}$ is the binomial coefficient and

$$e_k(T_1, \ldots, T_N) = \sum_{1 \le j_1 < j_2 < \cdots < j_k \le N} T_{j_1} T_{j_2} \ldots T_{j_k} \tag{S.14}$$

is the elementary symmetric polynomial in the N variables $T_1, \ldots, T_N$, which is made of $\binom{N}{k}$ terms corresponding to all possible combinations of k variables T_j out of N.

Since $T_{j_1} T_{j_2} \ldots T_{j_k}$ equals to 1 if and only if the subset $\{j_1, j_2, \ldots, j_k\}$ of k variables is among the ensemble of particles detected in D1, the quantity $\langle e_k(T_1 \ldots, T_N) \rangle$ is the sum of all the probabilities p_n with $n \ge k$ multiplied by the number $\binom{n}{k}$ of possibilities to take k terms out of n. In terms of FCS, the symmetrised multivariate moments can therefore be written

$$m_k = \binom{N}{k}^{-1} \sum_{n=k}^{N} \binom{n}{k} p_n. \tag{S.15}$$

This result can be mathematically demonstrated as follows. In our case, the variables T_j are binary ($T_j \in \mathbb{Z}^2$) and idempotent ($T_j^2 = T_j$). Under these conditions, a multiplication law $e_1\, e_j = j\, e_j + (j+1)\, e_{j+1}$ holds true. Using this multiplication law, one can prove the identity $e_k = \binom{e_1}{k}$ by induction. As $e_1 = \sum_{j=1}^{N} T_j = n$, the elementary symmetric polynomial e_k is simply equal to the binomial coefficient $\binom{n}{k}$. The symmetrised multivariate moment

defined as $m_k = \binom{N}{k}^{-1} \langle e_k \rangle$ writes

$$m_k = \binom{N}{k}^{-1} \left\langle \binom{n}{k} \right\rangle = \binom{N}{k}^{-1} \sum_{n=k}^{N} \binom{n}{k} p_n \tag{S.16}$$

as anticipated above, the average being replaced by its definition in terms of probabilities. We also obtain the relation

$$m_k = \frac{\langle (n)_k \rangle}{(N)_k} \tag{S.17}$$

where $(n)_k = n(n-1)\dots(n-k+1)$ is the falling factorial and $\langle (n)_k \rangle$ is called the factorial moment of order k.

Similar to Eq. (S.13), we define the symmetrised k-th order multivariate cumulants

$$\kappa_k = \binom{N}{k}^{-1} \langle\!\langle e_k(T_1 \dots T_N) \rangle\!\rangle. \tag{S.18}$$

When the system is fully symmetric under permutations, all multivariate moments and cumulants of k-th order are the same, and equal to the symmetrised quantities $m_k = \langle T_1 T_2 \dots T_k \rangle$ and $\kappa_k = \langle\!\langle T_1 T_2 \dots T_k \rangle\!\rangle$. In this case of full statistical equivalence between the particles, the relation between the symmetric multivariate moments m_k and cumulants κ_k are exactly the same as between the regular univariate moments $\langle n^k \rangle$ and cumulants $\langle\!\langle n^k \rangle\!\rangle$. Hence the standard combinatorial formula Eq. (S.11) applies

$$\kappa_k = \sum_{j=1}^{k} (j-1)!(-1)^{j-1} B_{kj}\left(m_1, m_2, \dots, m_{k-j+1}\right) . \tag{S.19}$$

where B_{kj} are the partial Bell polynomials. For reference, we quote the explicit relations for the values of k involved in the experiment,

$$\kappa_1 = m_1 \tag{S.20a}$$
$$\kappa_2 = -m_1^2 + m_2 \tag{S.20b}$$
$$\kappa_3 = 2\,m_1^3 - 3\,m_2\,m_1 + m_3 \tag{S.20c}$$
$$\kappa_4 = -6\,m_1^4 + 12\,m_1^2\,m_2 - 4\,m_1\,m_3 - 3m_2^2 + m_4 \tag{S.20d}$$
$$\kappa_5 = 24\,m_1^5 - 60\,m_1^3\,m_2 - 5\,m_1\,m_4 - 10\,m_2\,m_3 + 20\,m_1^2\,m_3 + 30\,m_1\,m_2^2 + m_5 \tag{S.20e}$$

Together with Eq. (S.15), these relations are used to compute symmetrised multivariate cumulants form the experimental counting statistics $p_n = P_{(N-n,n)}$.

We note that rescaling to spin variables, $s_i = 2\,T_i - 1$, transforms the multivariate cumulants as $\langle\!\langle s_j \rangle\!\rangle = 2\langle\!\langle T_j \rangle\!\rangle - 1$ and $\langle\!\langle s_1 \dots s_k \rangle\!\rangle = 2^k \langle\!\langle T_1 \dots T_k \rangle\!\rangle$ for $k \geq 2$, hence $\kappa_{k>1}$ can also be interpreted as irreducible spin correlation functions.

5.3. Comparison of different types of cumulants

One can also define factorial cumulants $\langle\!\langle n^k \rangle\!\rangle_{\mathrm{F}}$ [13, 14] whose generating function is $\ln\langle e^{zn} \rangle = \sum_{k=1}^{\infty} \langle\!\langle n^k \rangle\!\rangle_{\mathrm{F}} (z-1)^k / k!$ (expansion of the univariate cumulant generating function around $z = 1$). The corresponding factorial moments, $\langle (n)_k \rangle$, are simply averages of

the falling factorial [14]. The relations between these factorial cumulants $\langle\!\langle n^k \rangle\!\rangle_{\mathrm{F}}$ and the factorial moments $\langle (n)_k \rangle = \langle n!/(n-k)! \rangle$ are the same as between univariate cumulants and univariate moments in Eq. (S.10) and (S.11). The advantage of factorial cumulants is evident in a Poisson distribution, where all $\langle\!\langle n^k \rangle\!\rangle_{\mathrm{F}} = 0$ for $k > 1$.

We note that the symmetrised multivariate cumulant κ_k is *not* the factorial cumulant $\langle\!\langle n^k \rangle\!\rangle_{\mathrm{F}}$ of the FCS, because of the factor $(N)_k$ in the relation (S.17) between the symmetrised multivariate moment m_k and the factorial moment $\langle (n)_k \rangle$.

The difference between regular univariate cumulants $\langle\!\langle n^k \rangle\!\rangle$, factorial cumulants $\langle\!\langle n^k \rangle\!\rangle_{\mathrm{F}}$ and symmetrised multivariate cumulants κ_k can also be understood in terms of reference probability distributions. Namely, $\langle\!\langle n^k \rangle\!\rangle = 0$, $\langle\!\langle n^k \rangle\!\rangle_{\mathrm{F}} = 0$ and $\kappa_k = 0$ (for $k \geq 2$) are true for Gaussian (in continuous n limit), Poisson and binomial distributions, respectively.

6. ANALYSIS OF 2e/2e PARTITIONING DATA

In Fig. 2b, four electrons are sent such that the electrons are placed in pairs in two adjacent SAW minima (configuration denoted as 2e/2e and $N = 4$). This situation is used to explore the case of a less symmetric configuration, with non-equivalent same-order cumulants.

Similar to the case of one electron per minimum analysed in Fig. 2a, we can check from the counting statistics that electrons in different SAW minima remain uncorrelated. This is done by reconstructing the 2e/2e partitioning probabilities ($N = 4$) from the 2e partitioning data where only one minimum is filled with two electrons ($N = 2$), using the convolution of corresponding probability distributions

$$\begin{aligned} P_{(4,0)} &= P_{(2,0)}^2 \\ P_{(3,1)} &= 2P_{(1,1)}P_{(2,0)} \\ P_{(2,2)} &= P_{(1,1)}^2 + P_{(2,0)}P_{(0,2)} \\ P_{(1,3)} &= 2P_{(1,1)}P_{(0,2)} \\ P_{(0,4)} &= P_{(0,2)}^2 . \end{aligned} \tag{S.21}$$

The measured and reconstructed probabilities are shown in Fig. S8 and are in good agreement, confirming the absence of inter-SAW-minima correlations.

Further, we use this 2e/2e case (with interactions restricted to the electron pairs occupying the same minimum) to illustrate how higher-order multivariate cumulants can be calculated in case of partial permutational symmetry. The general formulas [12] expressing the relations between the multivariate moments $\langle T_{j_1}^{i_1} T_{j_2}^{i_2} \dots T_{j_l}^{i_l} \rangle$ and the multivariate cumulants can be summarised as follows [15]

$$\langle\!\langle T_{j_1}^{i_1} \dots T_{j_l}^{i_l} \rangle\!\rangle = \sum_{\{\mathcal{P}_l\}} (|\mathcal{P}_l\,| - 1)!\; (-1)^{|\mathcal{P}_l\,| - 1} \sum_{B \in \mathcal{P}_l} \Big\langle \prod_{r \in B} T_{j_r}^{i_r} \Big\rangle , \tag{S.22}$$

where $\{\mathcal{P}_l\}$ denotes the list of all partitions of a set $\{1, 2, \dots, l\}$ for a total of l variables. $B \in \mathcal{P}_l$ is a block in a partition $\mathcal{P}_l$, and $|\mathcal{P}_l\,|$ counts the number of blocks in that partition. In our case, we only consider cumulants $\langle\!\langle T_{j_1} T_{j_2} \dots T_{j_l} \rangle\!\rangle$ as the variables are idempotent ($T_j^2 = T_j$).

While general formulas are always valid, they involve too many different cumulants $\langle\!\langle T_{j_1} T_{j_2} \dots T_{j_l} \rangle\!\rangle$ for all to be determined by the partitioning data. However, symmetry considerations may lead to significant reduction in the number of non-equivalent multivariate functions. For complete permutational symmetry (when all electrons are statistically indistinguishable, e.g., all placed either in different or in the same SAW minimum), the cumulants κ_k are completely determined by counting statistics p_n, as described in Methods and Supplementary Note 5. In particular, Eq. (S.22) leads to Eq. (S.19) with Eq. (S.17) in this case. For systems that are not fully symmetric under permutations, not all multivariate cumulants of k-th order are equal in general and Eq. (S.19) for calculating the symmetrised cumulants κ_k from the symmetrised moments m_k is no longer applicable (however, the cumulants κ_k are always defined as in Eq. (S.18)).

Here we examine a specific case with partial symmetry which follows from considering the particular placement of the electrons in the SAW minima. In the present example of 2e/2e electrons, the interaction strength is different between the electrons in the same SAW minimum and the electrons in separate minima. We index electrons such that {1,2} corre-

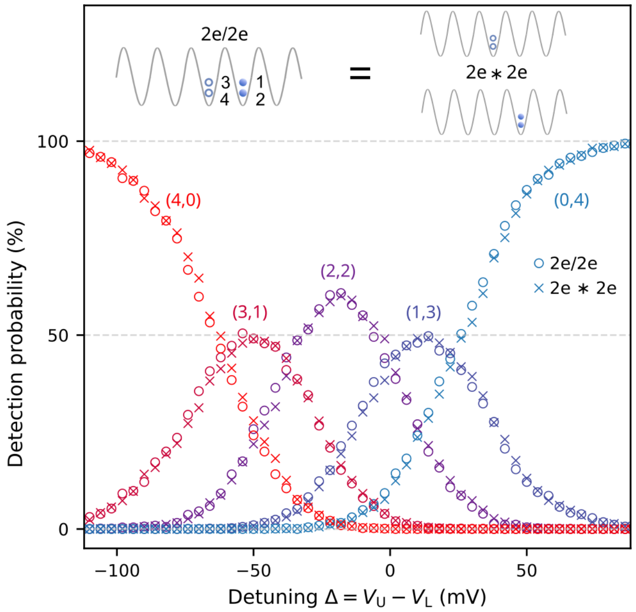

Fig. S8. **Experimental reconstruction of 2e/2e** ($N = 4$) **from 2e** ($N = 2$)**.** Detection probabilities for the partitioning of four electrons in a configuration with two electrons in each of two adjacent minima (2e/2e) compared to its reconstruction calculated from the detection probabilities measured from the partitioning of two interacting electrons in a single minimum (2e) using Eq. (S.21). Each data point is obtained from 3,000 single-shot measurements.

sponds to the pair in one minimum (denoted with $\bullet$) and $\{3,4\}$ to the other pair (denoted with $\circ$). There are only two non-equivalent second-order multivariate cumulants, one for electrons in the same minimum $\langle\langle T_1T_2\rangle\rangle = \langle\langle T_3T_4\rangle\rangle = \langle\langle \bullet\bullet\rangle\rangle$ and one for electrons in different minima $\langle\langle T_1T_3\rangle\rangle = \langle\langle T_1T_4\rangle\rangle = \langle\langle T_2T_3\rangle\rangle = \langle\langle T_2T_4\rangle\rangle = \langle\langle \bullet\circ\rangle\rangle$. Hence, following the definition (S.18), κ_2 is expressed as $\kappa_2 = (2\langle\langle \bullet\bullet\rangle\rangle + 4\langle\langle \bullet\circ\rangle\rangle)/6$. The third-order multivariate cumulants $\langle\langle T_aT_bT_c\rangle\rangle = \langle\langle \bullet\bullet\circ\rangle\rangle = \langle\langle \bullet\circ\circ\rangle\rangle = \kappa_3$ are all equal, as the subsystem remains physically equivalent for selection of any subset of three electrons (two electrons in the same minimum and one in another). Therefore, symmetrised κ_3 can be used for any third-order cumulant in Eq. (S.22).

Using the above partial symmetry conditions and Eq. (S.15) and (S.22), we express partitioning probabilities p_n via multivariate cumulants (Table S2). With two different second-order cumulants, the system is undetermined. However, based on the reconstruction of 2e/2e ($N = 4$) probability distribution from 2e ($N = 2$) partitioning data, we have confirmed that there is no inter-SAW-minima correlation, $\langle\langle \bullet\circ\rangle\rangle = 0$. This condition allows us to compute the cumulants using the expressions in Table S2 by setting $\langle\langle \bullet\circ\rangle\rangle = 0$. In this case $\kappa_2 = \langle\langle \bullet\bullet\rangle\rangle/3$. The symmetrised cumulants κ_2, κ_3 and κ_4 computed in this way are depicted in Fig. 2e of the main text. One can see that κ_3 and κ_4 are close to zero, demonstrating the absence of higher than second-order correlations.

Alternatively, we could have assumed that $\kappa_4 = 0$ and then computed $\langle\langle \bullet\bullet\rangle\rangle$ and $\langle\langle \bullet\circ\rangle\rangle$ separately (with the expectation that the latter will be close to zero).

p_n	κ_1	κ_1^2	κ_1^3	κ_1^4	$\langle\langle\bullet\bullet\rangle\rangle$	$\langle\langle\bullet\circ\rangle\rangle$	$\kappa_1\langle\langle\bullet\bullet\rangle\rangle$	$\kappa_1\langle\langle\bullet\circ\rangle\rangle$	$\kappa_1^2\langle\langle\bullet\bullet\rangle\rangle$	$\kappa_1^2\langle\langle\bullet\circ\rangle\rangle$	$\langle\langle\bullet\bullet\rangle\rangle^2$	$\langle\langle\bullet\circ\rangle\rangle^2$	κ_3	$\kappa_1\,\kappa_3$	κ_4	1
p_0	-4	6	-4	1	2	4	-4	-8	2	4	1	2	-4	4	1	1
p_1	4	-12	12	-4	-4	-8	12	24	-8	-16	-4	-8	12	-16	-4	0
p_2	0	6	-12	6	2	4	-12	-24	12	24	6	12	-12	24	6	0
p_3	0	0	4	-4	0	0	4	8	-8	-16	-4	-8	4	-16	-4	0
p_4	0	0	0	1	0	0	0	0	2	4	1	2	0	4	1	0

Table S2. **Probabilities and cumulants for the 2e/2e case ($N = 4$).** Coefficients for expressing the probabilities $p_n = P_{(N-n,\,n)}$ from the multivariate cumulants, taking into account the symmetry but without assumption about the strength of the intra-minimum versus inter-minimum interaction. p_n is the sum of the terms in the top row with each term multiplied by its corresponding coefficient.

REFERENCES

[1] S. Takada, H. Edlbauer, H. V. Lepage, J. Wang, P. A. Mortemousque, G. Georgiou, C. H. Barnes, C. J. Ford, M. Yuan, P. V. Santos, *et al.*, Sound-driven single-electron transfer in a circuit of coupled quantum rails, Nature Communications **10**, 4557 (2019).

[2] J. Wang, H. Edlbauer, A. Richard, S. Ota, W. Park, J. Shim, A. Ludwig, A. D. Wieck, H. S. Sim, M. Urdampilleta, *et al.*, Coulomb-mediated antibunching of an electron pair surfing on sound, Nature Nanotechnology **18**, 721 (2023).

[3] U. Weiss and W. Haeffner, Complex-time path integrals beyond the stationary-phase approximation: decay of metastable states and quantum statistical metastability, Physical Review D **27**, 2916 (1983).

[4] U. Weiss, *Quantum dissipative systems* (World Scientific, 2012).

[5] P. Hänggi, P. Talkner, and M. Borkovec, Reaction-rate theory: fifty years after Kramers, Reviews of Modern Physics **62**, 251 (1990).

[6] I. Affleck, Quantum-statistical metastability, Physical Review Letters **46**, 388 (1981).

[7] S. Birner, T. Zibold, T. Andlauer, T. Kubis, M. Sabathil, A. Trellakis, and P. Vogl, Nextnano: general purpose 3-D simulations, IEEE Transactions on Electron Devices **54**, 2137 (2007).

[8] L. Fricke, M. Wulf, B. Kaestner, V. Kashcheyevs, J. Timoshenko, P. Nazarov, F. Hohls, P. Mirovsky, B. Mackrodt, R. Dolata, *et al.*, Counting statistics for electron capture in a dynamic quantum dot, Physical Review Letters **110**, 126803 (2013).

[9] L. S. Levitov, H. Lee, and G. B. Lesovik, Electron counting statistics and coherent states of electric current, Journal of Mathematical Physics **37**, 4845 (1996).

[10] L. Comtet, *Advanced Combinatorics* (Springer Dordrecht, 1974).

[11] G. S. James and A. J. Mayne, Cumulants of functions of random variables, Sankhyā: The Indian Journal of Statistics, Series A (1961-2002) **24**, 47 (1962).

[12] C. W. Gardiner, Handbook of stochastic methods - for physics, chemistry and the natural sciences, second edition, in *Springer Series in Synergetics* (1986).

[13] D. Kambly, C. Flindt, and M. Büttiker, Factorial cumulants reveal interactions in counting statistics, Physical Review B **83**, 075432 (2011).

[14] M. Kitazawa and X. Luo, Properties and uses of factorial cumulants in relativistic heavy-ion collisions, Physical Review C **96**, 024910 (2017).

[15] S. Floerchinger and U. A. Wiedemann, Statistics of initial density perturbations in heavy ion collisions and their fluid dynamic response, Journal of High Energy Physics **2014**, 5 (2014).
